# Estimating funds required for UHC within Indian States

**DOI:** 10.1016/j.lansea.2023.100165

**Published:** 2023-02-23

**Authors:** Nachiket Mor, Sudheer Kumar Shukla

**Affiliations:** a Banyan Academy of Leadership in Mental Health, India; b Centre for the Study of Regional Development, School of Social Sciences, Jawaharlal Nehru University, India

**Keywords:** UHC, Financing, Health finance, Healthcare costs

## Abstract

**Background:**

Universal Health Coverage (UHC) has been high on national and international agendas since its adoption as one of the Sustainable Development Goals (SDGs). Within India, there is a wide variation in the total amounts per capita spent by each state government (Government Health Expenditure or GHE) on healthcare. Bihar, with a GHE of 556 per capita (per annum), has the lowest state government spending, but there are many states in which governments spend more than four times that amount on a per capita basis. However, despite this, no state offers UHC to its residents. This failure to provide UHC could be because even the highest amounts spent by the state governments are too low for them to offer UHC or because the cost differences between states are very high. It is also possible, however, that a poor design of the government-owned health system and the degree of waste embedded within it could account for this. It is important to understand which of these factors is responsible because it then provides a clue as to what the best path to UHC might be in each state.

**Methods:**

One way to do that would be to arrive at one or more broad estimates of the amounts needed to finance UHC and to compare them with actual amounts being spent by the governments in each state. Older research provides two such estimates. In this paper, using secondary data, we add to them using four additional approaches so that we can build greater confidence in the estimation of amounts needed by each state to offer UHC to its residents. We refer to these as *Outside-in*, *Actuarial*, *Normative*, and *Inside-Out*.

**Findings:**

We find that, with the exception of the approach which assumes that the current design of the government health system is optimal and only needs added investment to offer UHC (the *Inside-out* approach), all the other approaches give a value of between 1302 and 2703 per capita for UHC, with 2000 per capita providing a *reasonable* point estimate. We also find no evidence to support the view that these estimates are likely to vary between states.

**Interpretation:**

These results suggest that several Indian states may have an inherent ability to offer UHC with government financing alone and that a high degree of waste and inefficiency in the manner in which government funds are currently being deployed may well be behind their apparent inability to do so already. Another implication of these results is that several states may also be further away from the goal of offering UHC than an initial analysis of their GHE as a proportion of their Gross State Domestic Product (GSDP), i.e., GHE/GSDP, may suggest. Of particular concern are the states of Bihar, Jharkhand, Madhya Pradesh, and Uttar Pradesh, all of which have GHE/GSDP greater than 1%, but because their absolute levels of GHE are well below 2000, in order to reach UHC, they may need to more than triple their annual health budgets.

**Funding:**

The 10.13039/501100005918Christian Medical College Vellore supported the second author (Sudheer Kumar Shukla) through a grant from the 10.13039/501100007296Infosys Foundation. Neither of these two entities had any role in the study design, data collection, data analysis, interpretation, writing of the manuscript, or the decision to submit it for publication.

**Research in context**

**Evidence before this study**

Estimating the costs associated with UHC has been attempted globally using several approaches. In the Indian context, two attempts have used a fixed set of diseases (benefits package) and have estimated the costs associated with addressing them using standard treatment guidelines.

**Added value of this study**

We attempt to add to these estimates in the study by using four additional approaches. These are described below:

Outside-In Approach: This attempts to arrive at the amount required per capita for UHC by comparing state-level government health expenditures with global benchmarks using different estimates of the rupee–dollar exchange rate. It implicitly defines UHC using standards set by developing countries such as Colombia, Jordan, Nicaragua, Peru, and Thailand, which have built high-performing health systems at modest costs.
Actuarial Approach: This takes costs associated with different levels of care and their utilisation rates to arrive at a range of possible per capita UHC costs. It implicitly defines UHC as the full provision of primary, secondary, and tertiary care at different utilisation levels.
Normative Approach: This uses the 2022 Indian Public Health Standards (IPHS) and estimates the amounts required to meet them to arrive at per capita UHC costs. Here the implicit definition of UHC is the universal availability of primary, secondary, and tertiary healthcare services as defined by the 2022 IPHS standards.
Inside-Out Approach: This approach assumes that the designs of Indian government-owned health systems and tax-financed insurance programs are already optimal and seeks to estimate the amounts needed to fill the gaps between UHC and the current government expenditures. Here the implicit assumption is that only the funding available to the public sector needs to be expanded to offer UHC without making any other changes in its design.

These four estimates add to the two previously arrived at by other researchers allowing us to develop a high degree of confidence in our efforts to arrive at the amounts needed to offer UHC within each Indian state.

**Implications of all the available evidence**

All the available evidence taken together suggests that, except for the approach which assumes that the current design of the government health system is optimal and only needs added investment to offer UHC (the Inside-out approach), all other methods, at 2018 prices, give an estimate of between Rs. 1302 and Rs 2703 per capita for UHC with Rs 2000 per capita providing a reasonable point estimate. The per capita point estimate of Rs 2000 implies that several Indian states may have an inherent ability to offer UHC with government financing alone and that a high degree of waste and inefficiency in the manner in which government funds are currently being deployed may well be behind their apparent inability to do so already, particularly in states where government health expenditures already exceed Rs.2000 per capita.

Another implication of these results is that several states may be further away from the goal of offering UHC than an initial analysis of their government health expenditures (GHE) as a proportion of their Gross State Domestic Product (GSDP), i.e., GHE/GSDP, may suggest. Of particular concern are the states of Bihar, Jharkhand, Madhya Pradesh, and Uttar Pradesh, all of which have GHE/GSDP greater than 1%, but because their absolute levels of GHE are well below Rs. 2000, to reach UHC, they may need to more than triple their annual health budgets.

## Introduction

Universal Health Coverage (UHC) has been high on national and international agendas since its adoption as one of the Sustainable Development Goals (SDGs). However, the goal lacks sufficient clarity, in part because it has proven difficult to specify what UHC means with any degree of precision and estimate the resource requirements associated with it. Globally, studies have adopted several approaches to estimate these resource requirements. These include a “step-down” approach in Cambodia,^1^ an actuarial model for the costing of UHC in Armenia,^2^ a bottom-up approach for the allocation of resources in Malawi,^3^ and the combination of a bottom-up approach and the Disease Control Priorities cost model^4^ for resource-constrained low and middle-income countries. In the case of India, a top-down approach and a combination of bottom-up and top-down approaches have been used.^5^, ^6^, ^7^

Within India, there is also a wide variation in the total amounts spent by the state governments (Government Health Expenditure or GHE) on healthcare. Bihar, with a GHE of 556 per capita (per annum), has the lowest state government spending, but there are many states in which governments spend more than four times that amount on a per capita basis (see Table A7 in National Health Accounts for 2017–18^8^). However, despite this, with high out-of-pocket expenditures (see Table A6 in National Health Accounts for 2017–18^8^), poor health outcomes (see National Family Health Survey 2019–20^9^ and Table 10 in Mor^10^), and low responsiveness and quality of care,^13^, ^14^, ^15^, ^16^, ^17^, ^18^ it is clear that no state offers UHC to its residents.

**Table 1:** Exchange rates implied by the relative costs of major surgeries in USA & India (adapted from Reddy and Qadeer^11^).

Medical procedure	Procedure cost		Implied exchange
	USA ($)	India ()	Rate (/$)
Heart bypass	130,000	799,100	6.15
Heart valve replacement	160,000	719,190	4.49
Angioplasty	57,000	879,010	15.42
Hip replacement	43,000	719,190	16.73
Hysterectomy	20,000	239,730	11.99
Knee replacement	40,000	679,235	16.98
Spinal fusion	62,000	439,505	7.09

**Table 2:** Simulation of government health expenditures.

State	State GHE as % of			State GHE in PPP$ at			
	State	State	National	(/$)			
	GGE	GSDP	GDP	2	5	20	80
India	5.1	1.4%	1.4%	$877	$351	$88	$22
**Basic Healthcare**							
Bihar	5.0	1.4%	0.4%	$278	$111	$28	$7
Jharkhand	4.7	1.1%	0.6%	$401	$160	$40	$10
Uttar Pradesh	5.8	1.2%	0.6%	$401	$160	$40	$10
Madhya Pradesh	4.9	1.1%	0.8%	$490	$196	$49	$12
**Moderate UHC**							
Punjab	5.0	0.7%	0.8%	$543	$217	$54	$14
West Bengal	6.5	1.0%	0.8%	$544	$218	$54	$14
Odisha	5.7	1.2%	0.9%	$604	$241	$60	$15
Maharashtra	6.1	0.7%	1.0%	$678	$271	$68	$17
Rajasthan	6.3	1.2%	1.1%	$685	$274	$68	$17
Andhra Pradesh	5.3	0.9%	1.1%	$691	$276	$69	$17
Assam	7.5	1.6%	1.1%	$696	$278	$70	$17
Haryana	4.6	0.6%	1.1%	$714	$286	$71	$18
Karnataka	5.5	0.7%	1.1%	$738	$295	$74	$18
Gujarat	7.0	0.8%	1.2%	$751	$300	$75	$19
Chhattisgarh	6.4	1.5%	1.2%	$758	$303	$76	$19
Tamil Nadu	6.5	0.8%	1.2%	$811	$324	$81	$20
Uttarakhand	5.1	0.8%	1.3%	$813	$325	$81	$20
Jammu and Kashmir	4.3	1.6%	1.3%	$840	$336	$84	$21
Telangana	5.7	0.8%	1.3%	$849	$340	$85	$21
**Full UHC**							
Tripura	6.6	1.8%	1.5%	$997	$399	$100	$25
Manipur	5.6	2.5%	1.5%	$1004	$401	$100	$25
Kerala	7.3	1.1%	1.7%	$1136	$454	$114	$28
Nagaland	5.4	2.5%	2.4%	$1538	$615	$154	$38
Himachal Pradesh	7.2	1.6%	2.4%	$1589	$635	$159	$40
Delhi	18.5	1.0%	2.8%	$1800	$720	$180	$45
Sikkim	8.2	2.0%	3.6%	$2330	$932	$233	$58
Goa	7.7	1.4%	3.7%	$2428	$971	$243	$61
Meghalaya	15.8	4.8%	3.8%	$2472	$989	$247	$62
Puducherry	8.5	1.6%	4.0%	$2625	$1050	$263	$66
Mizoram	7.6	3.6%	5.2%	$3385	$1354	$339	$85
Arunachal Pradesh	6.7	4.2%	7.3%	$4725	$1890	$473	$118

**Table 3:**
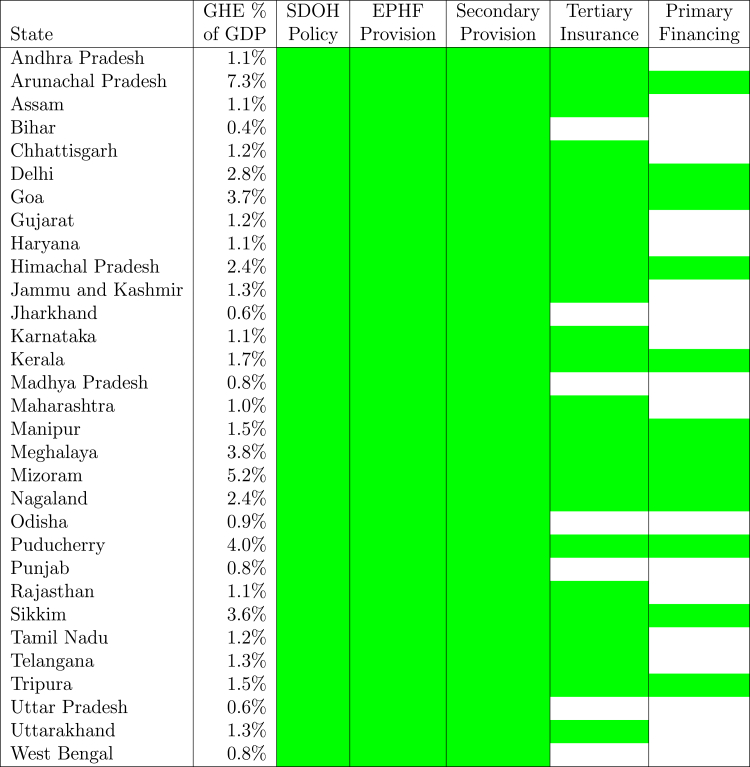
Potential UHC pathway for each state.

**Table 4:** Simulated actuarial costing.

	Min			Mid			Max		
	P	S	T	P	S	T	P	S	T
Incidence Rate	200%	1%	0.10%	250%	2%	0.20%	300%	3%	0.30%
Patients/Unit	12,500	60	30	10,000	50	25	7500	40	20
Capital Cost (lac)/Unit	2.0	7.5	25.0	4.0	10.0	30.0	6.0	12.5	35.0
Replacement Life (in years)	7.5	10.0	12.5	5.0	7.5	10	2.5	5.0	7.5
Revenue ( ′000)/Patient	0.05	5	40	0.10	10	50	0.15	15	60
Admin Charge (%)	10%	5%	2.5%	15%	7.5%	5%	20%	10%	7.5%

Per Capita UHC Cost	114	65	48	308	268	129	636	683	264
Per Capita Total UHC Cost	227			705			1582		
Total UHC Cost (crore)	29,501			91,628			205,660

**Table 5:** PMJAY claims data analysis (adapted from Table 2 in Dong and colleagues^12^).

Healthcare Level	Claim Amount		Incidence Rate	
	From	To	per 100,000	per 100
			Rate	%
Secondary	<30,000	30,000	1307.7	1.3077%
Tertiary	30,000	100,000	220.8	0.2208%
	100,000	500,000	36.9	0.0369%
	500,000	>500,000	0.4	0.0004%

**Table 6:** Normative total budget per capita.

Level of Healthcare Facility (Level)	Total facilities required (Number)	Beds/Facility (Number)	HR expenses/capita ()	Infrastructure + consumables expenditure/capita ()	Total expenditure/capita ()
Medical College (MC)	825	900	201	854	1056
District Hospital (DH)	993	500	135	320	455
Sub Divisional Hospital (SDH)	1211	100	55	53	108
Community Health Centre (CHC)	10,865	100	454	266	720
Primary Health Centre (PHC)	47,289	n/a	315	105	420
Sub Centre (SC)	255,658	n/a	234	294	528
All			1394	1892	3286

**Table 7:** Inside-out estimate of expenditure needed for UHC.

Expenditure (Category)	Existing government expenditure (crore)	Required additional government expenditure (crore)	Total expenditure (crore)	Total expenditure/capita ()
Insurance (Government + Private)	28,067	97,858	125,925	960
Government Direct	231,104		231,104	1762
Private Out-Patient OOPE		192,729	192,729	1469
Private In-Patient OOPE		115,548	115,548	881
For Unmet Need of Household		36,130	36,130	275
For Other Additional Household healthcare		26,801	26,801	204
Needed for UHC (India)	259,171	469,066	728,237	5552

**Table 8:** Estimated UHC costs.

Estimation Approach	Per Capita UHC Cost Estimate (/capita)	Estimation Year (year)	Inflation Adjusted @ 5% to 2018 (/capita)
Outside-In (Table 2)	2000	2022	1645
Actuarial (Table 4)	1582	2022	1302
Normative (Table 6)	3286	2022	2703
Inside-out (Table 7)	5552	2018	5552
Prinja and colleagues^5^	1713	2012	2296
Bhatt and colleagues^6^	1626	2014	1976

**Table 9:** Additional funds needed for UHC with 2000 per capita as the target GHE.

State	GSDP crore	GGE crore	GHE	Gap		Target			Target		Target
			(Per Capita)		GHE	GHE	Gap	GHE	GHE	GHE	GHE
					(crore)			(% of GSDP)		(% of GGE)	
Andhra Pradesh	793,186	134,704	1381	619	7181	10,400	3219	0.91%	1.31%	5.33%	7.72%
Arunachal Pradesh	22,432	14,089	9450	–	945	945	–	4.21%	4.21%	6.71%	6.71%
Assam	288,691	63,174	1392	608	4733	6800	2067	1.64%	2.36%	7.49%	10.76%
**Bihar**	**484,740**	**131,531**	**556**	**1444**	**6561**	**23,600**	**17,039**	**1.35%**	**4.87%**	**4.99%**	**17.94%**
Chhattisgarh	284,194	66,231	1516	484	4245	5600	1355	1.49%	1.97%	6.41%	8.46%
Delhi	686,824	36,997	3599	–	6838	6838	–	1.00%	1.00%	18.48%	18.48%
Goa	70,494	12,637	4855	–	971	971	–	1.38%	1.38%	7.68%	7.68%
Gujarat	1,328,068	144,373	1502	498	10,063	13,400	3337	0.76%	1.01%	6.97%	9.28%
Haryana	649,592	86,795	1428	572	3998	5600	1602	0.62%	0.86%	4.61%	6.45%
Himachal Pradesh	138,351	30,809	3177	–	2224	2224	–	1.61%	1.61%	7.22%	7.22%
Jammu and Kashmir	137,427	51,269	1679	321	2183	2600	417	1.59%	1.89%	4.26%	5.07%
**Jharkhand**	**276,243**	**62,903**	**801**	**1199**	**2964**	**7400**	**4436**	**1.07%**	**2.68%**	**4.71%**	**11.76%**
Karnataka	1,357,579	173,149	1476	524	9594	13,000	3406	0.71%	0.96%	5.54%	7.51%
Kerala	701,577	108,697	2272	–	7952	7952	–	1.13%	1.13%	7.32%	7.32%
**Madhya Pradesh**	**724,729**	**161,159**	**980**	**1020**	**7938**	**16,200**	**8262**	**1.10%**	**2.24%**	**4.93%**	**10.05%**
Maharashtra	2,411,600	268,413	1356	644	16,408	24,200	7792	0.68%	1.00%	6.11%	9.02%
Manipur	23,835	10,702	2007	–	602	602	–	2.53%	2.53%	5.63%	5.63%
Meghalaya	30,790	9406	4943	–	1483	1483	–	4.82%	4.82%	15.77%	15.77%
Mizoram	18,737	8877	6770	–	677	677	–	3.61%	3.61%	7.63%	7.63%
Nagaland	24,492	11,466	3075	–	615	615	–	2.51%	2.51%	5.36%	5.36%
Odisha	434,769	92,946	1207	793	5311	8800	3489	1.22%	2.02%	5.71%	9.47%
Puducherry	32,962	6201	5250	–	525	525	–	1.59%	1.59%	8.47%	8.47%
Punjab	478,636	64,817	1086	914	3258	6000	2742	0.68%	1.25%	5.03%	9.26%
Rajasthan	835,170	166,465	1369	631	10,404	15,200	4796	1.25%	1.82%	6.25%	9.13%
Sikkim	23,495	5659	4660	–	466	466	–	1.98%	1.98%	8.23%	8.23%
Tamil Nadu	1,461,841	188,077	1621	379	12,158	15,000	2843	0.83%	1.03%	6.46%	7.98%
Telangana	753,127	109,267	1698	302	6283	7400	1117	0.83%	0.98%	5.75%	6.77%
Tripura	44,219	12,134	1993	7	797	800	3	1.80%	1.81%	6.57%	6.59%
**Uttar Pradesh**	**1,460,443**	**305,311**	**801**	**1199**	**17,782**	**44,400**	**26,618**	**1.22%**	**3.04%**	**5.82%**	**14.54%**
Uttarakhand	222,836	34,997	1625	375	1788	2200	413	0.80%	0.99%	5.11%	6.29%
West Bengal	999,585	160,445	1088	912	10,445	19,200	8755	1.04%	1.92%	6.51%	11.97%
India	17,118,815				231,104	334,812	103,708	1.35%	1.96%		

The bold text refers to states which have per capita GHE below Rs.1000 and are, as a result, the furthest away from the amount of Rs.2000 GHE per capita required for UHC.

**Table 10:** UHC funds needed with different per capita targets.

Target GHE per capita ()	Additional funds needed for UHC (crore)	Target GHE (% GDP)	Total funds needed for UHC (crore)
1500	44,023	1.61%	275,128
**2000**	**103,708**	**1.96%**	**334,812**
2500	166,604	2.32%	397,708
3000	230,000	2.70%	461,558

The bold text refers to the funds needed for UHC at the point estimate of Rs.2000 GHE per capita.

This failure to provide UHC could be because even the highest amounts spent by the state governments are too low for them to offer UHC or because the cost differences between states are very high. It is also possible, however, that a poor design of the government-owned health system and the degree of waste embedded within it could account for this. It is important to understand which of these factors is responsible because it then provides an indication of what the best path to UHC might be in each state. One way to do that would be to arrive at one or more broad estimates of the amounts needed to finance UHC and to compare them with actual amounts being spent by the governments in each state. Older research provides two such estimates. In this paper, we add to them using four different approaches so that we can build greater confidence in the estimation of amounts needed by each state to offer UHC to its residents.

## Methods

In one of the earliest such efforts, Prinja and colleagues^5^ defined UHC as the cost of treating the existing burden of disease in India using standard treatment protocols and the existing mix of public and private providers. It arrived at an estimate of 1713 per capita per year. Following the publication of the report of the High-Level Expert Group (HLEG) on UHC in 2011,^19^ using a detailed consultative and analytical process, Bhatt and colleagues^6^ made another attempt at estimating the optimal costs associated with offering UHC in India. An essential health package (EHP) of 80 services was first arrived at under four broad heads and 34 categories (see Fig. 3 in Bhatt and colleagues^6^), and UHC was defined as providing comprehensive treatment for the EHP. The most cost-effective care pathways for these services were then chosen, and the costs associated with providing them at all levels of the health system were estimated. These were aggregated to arrive at an estimate of 1626 per capita as the costs associated with the provision of UHC (see p 59 of Bhatt and colleagues^6^).

Given the inherent imprecision associated with the definition of UHC, in this paper, instead of repeating these two older exercises,^5^^,^^6^ four additional approaches, each with its own implicit definition of UHC, are considered to arrive at additional estimates of per-capita expenditures associated with the offering of UHC. These six estimates are then used to arrive at a plausible range of values for the cost of UHC in India with the view that the *true cost* lies within this range.

1. Outside-In Approach: This attempts to arrive at the amount required per capita for UHC by comparing state-level government health expenditures with global benchmarks using different estimates of the Rupee–Dollar exchange rate. It implicitly defines UHC using standards set by developing countries such as Colombia, Jordan, Nicaragua, Peru, and Thailand, which have built high-performing health systems at modest costs (see Tables 4 and 5 in Mor^10^).
2. Actuarial Approach: This takes costs associated with different levels of care and their probable utilisation rates to arrive at a range of possible per capita UHC costs. It implicitly defines UHC as the full provision of primary, secondary, and tertiary care at different levels of utilisation.
3. Normative Approach: This uses the 2022 Indian Public Health Standards (IPHS) and estimates amounts required to meet them to arrive at per capita UHC costs. Here the implicit definition of UHC is the universal availability of primary, secondary, and tertiary healthcare services as defined by the 2022 IPHS standards.
4. Inside-Out Approach: This approach starts with the assumption that the designs of Indian government-owned health systems and tax-financed insurance programs are already optimal and seeks to estimate the amounts needed to fill the gaps between UHC and the current government expenditures. Here the implicit assumption is that only the funding available to the public sector needs to be expanded in order to offer UHC without making any other changes in its design.

In the following paragraphs, the methodology behind these approaches is discussed in some detail.

### Outside-in approach

In this approach, an attempt is made to arrive at the amount required per capita for UHC by comparing state-level government health expenditures with global benchmarks using different estimates of the Rupee-Dollar Purchasing Power Parity (PPP$) exchange rate. The market Rupee–Dollar exchange rate is arrived at using relative prices of tradeable goods and services. These are often only subsets of the full sets of goods and services that are available in any country because several are not tradeable. Human resources, for example, are a key non-tradeable factor in India because a free global migration of Indian labour is not permitted.

Recognising this, the International Monetary Fund (IMF) publishes what are referred to as Purchasing Power Parity (PPP) exchange rates which attempt to find the value of a common basket of goods in each country and then, using the U.S. Dollar ($) as the common currency, arrive at these PPP$ exchange rates.^20^ These are the rates that are most often used in global comparisons. However, unlike, say, steel plants, which are very capital-intensive, health is highly labour-intensive. The IMF PPP$ exchange rate is an average that covers both of these sectors, but it is entirely possible that if health is looked at on its own, the PPP$ exchange rate could be very different.

The paper attempts to compute the PPP$ exchange rate for the health sector in India by looking at multiple benchmarks – the salaries of nurses and the prices of different procedures in the US and in India. Data for this analysis have been obtained from multiple published sources.^8^^,^^10^^,^^11^^,^^21^^,^^22^

### The actuarial approach

In this approach, healthcare costs are simulated using a range of *plausible* estimates of incidence rates and costs of infrastructure at different levels of care. Given the paucity of published data on this, these estimates have been arrived at by the first author based on discussions with healthcare providers and health insurers. Indirect validation of the estimates that have been used in this analysis is provided by the claims data from government insurance schemes (see Table 2 Dong and colleagues^12^) and commercial insurance premiums for comprehensive health insurance schemes.^23^ The different parameters that are used in the simulation exercise are defined below:

• Incidence Rate: Number of patients needing each level of care as a proportion of the total covered population.
• Patients/Unit: Number of patients that each primary care facility or each hospital bed can properly accommodate each year. For hospital beds, this is linked to the average length of stay – if a patient needs to stay in a hospital for, say, five days, then a single bed can, at most, accommodate only 60 patients in a year.
• Capital Cost (lac)/Unit: Capital cost of each primary care facility or each hospital bed.
• Replacement Life (in years): The number of years after which the primary care facility would need to be rebuilt or the hospital bed replaced – the inverse of this number is the rate at which the asset needs to be depreciated.
• Revenue (′000)/Patient: Total income per patient-visit that the primary care facility or hospital would expect to be paid to cover all its costs (other than capital recovery and administrative charge, which would be additional). The facility or hospital could be government-owned or privately owned.
• Admin Charge (%): The additional administrative charge that would need to be levied to cover administrative expenses.

### The normative approach

In this approach, the Indian Public Health Standards (IPHS) 2022^24^ have been used to arrive at an estimate of the cost of UHC in India. IPHS guidelines focus on the services to be provided at each level of health facility (except medical colleges). These form the basis for the norms for specified components of the health system, such as infrastructure, human resources, drugs, diagnostics and equipment, administrative and support services, quality assurance and improvement, and monitoring and supervision. Under infrastructure, a full list of components, such as parking, buildings, rooms, beds, counters, different units of health equipment, radiology units, operation theatres, blood and oxygen units, residential quarters, guest houses, mortuary, and toilets and sign boards, are included.

To arrive at the costs associated with these IPHS norms, the prices of equipment and units have been estimated at current (2022) market rates and were compiled from different sources, including private marketplaces such as IndiaMART and the government e-marketplace GeM. Spending on medicines, diagnostics, equipment, quality assurance, and clinical governance was also similarly estimated. Administrative personnel, specialists and medical officers, allied health professionals, other allied health professionals, pharmacists, staff nurses, cleaning staff, and other categories of staff have all been included in human resources. The salaries of personnel have been compiled from different websites such as AmbitionBox, Indeed, and Glassdoor. The numbers of doctors, staff, beds, operating rooms, and other infrastructure components required for each health facility, including Sub Centres (SCs), Health and Wellness Centres (HWCs), Primary Health Centres (PHCs), Community Health Centres (CHCs), and District Hospitals (DHs) and Sub-District Hospitals (SDHs), have been taken from IPHS 2022 guidelines.^24^

The calculations in the paper for each level of the healthcare facility are based on the norm for the highest number of beds associated with that level – 500 beds for a DH, 100 beds for an SDH, and 100 beds for a CHC. For PHCs and SCs, the IPHS standards do not stipulate a specific number of beds. The district-wise beds and numbers of health facilities required in the country have been estimated according to population norms with the help of the IPHS 2022 guidelines^24^ and Census 2011.^25^ Since there are no IPHS norms for this, the estimate of 825 Medical Colleges with 900 beds each has been based on the assumption that there is at least one Medical College in each district and the 2018 Medical Council of India guidelines.^26^ To compute per capita requirements, the projected Indian population for 2022 of 1,373,761,000 (137.4 crore; 1 crore ≡ 10 million; 1 lac ≡ 100,000) persons has been taken from the population projections for India and the states 2011–2036 report.^27^ Additional details on the data and the computation methods used can be found in the UHC Costing Data Supplement Normative Approach.

### The inside-out approach

This analysis starts with the assumption that the core design of the Indian health system and tax-financed health insurance programs is optimal. It estimates the added amounts needed to cover the uninsured population using the per-capita insurance premiums currently being paid by the government. The current per-capita out-of-pocket expenditures (OOPE) have been used to arrive at the additional amounts needed to serve those with unmet needs. The total needed for UHC then is the current set of expenditures by the government and private individuals, along with the added sums needed to cover those who are uninsured or have unmet needs for healthcare. The projected population for 2017-18 has been taken from population projections for India and States 2011–2036 report, which was 1,311,567,500 (131.2 crore; 1 crore ≡ 10 million; 1 lac ≡ 100,000) persons.^27^ The amounts needed to fill these gaps have been estimated in four subcategories of expenditure.

#### Government direct

This constitutes spending under all schemes funded and managed by the union, state, and local governments, including quasi-governmental organisations and donors (wherever funds are channelled through government organisations).

#### Government insurance

This includes funds allocated by the government towards payment of premiums for union and state government-financed health insurance schemes, employee benefit schemes, or any reimbursements made to government employees for healthcare purposes and social health insurance scheme expenditures. This indicates the extent of pooled funds available for specific categories of the population. The expenditure on social health insurance schemes and government-funded health insurance has been taken into account to calculate the government expenditure on currently ongoing insurance programs. In addition, the required amount for uninsured individuals has been estimated based on current government expenditures for covered beneficiaries.

#### Private OOPE and commercial insurance

This comprises health expenditures by households (consumers), which include direct out-of-pocket expenditures (OOPE) and private health insurance expenditures (indirect). Under OOPE, data for out-of-pocket expenditures for outpatient care in private institutions, out-of-pocket expenditures for inpatient care in private institutions, and required household expenditures for unmet healthcare needs have been obtained from the 75th round of National Sample Survey (NSS) of health consumption data (2017–18).^28^ For inpatient health expenditures, the average medical expenses incurred during hospital stay per hospitalisation case (excluding childbirth) are available for 365 days. For outpatient health expenditures, the average medical expenditure incurred per episode of illness (excluding inpatient cases) is available for 15 days, which has been scaled up to 365 days to obtain the annual number. The average inpatient and outpatient expenditures on treatment comprise different components such as doctor's fees, medications, diagnostic tests, bed charges, and other medical expenses, and transportation charges and other nonmedical expenses in the NSS data. Unit-level data are further aggregated from the case level to the individual level and used to calculate OOPE for both inpatient and outpatient care over a 365-day period.

OOPE has been estimated by deducting from total health expenditure the amount of health expenditure reimbursed by medical insurance or an employer. OOPE in government hospitals has been excluded from the estimate due to its subsidised nature, and therefore only OOPE in private health institutions has been considered. Thus, the expenditure for outpatient care or inpatient care at a private hospital by individuals from their own pocket has been denoted as private outpatient OOPE and private inpatient OOPE, respectively. Individuals or families who have had illnesses but have not been able to visit healthcare providers for various reasons (such as lack of medical facilities available in the neighbourhood, inability to pay, and excessive waiting time) have been considered to have unmet needs. The estimated expected expenditures for a patient with unmet needs have been calculated using the percentage of unmet needs, the prevalence, and the expenses of outpatient and inpatient cases. Private health insurance expenditures refer to expenses where households or employers pay an insurance premium to be covered under a specific health plan. These have been compiled from the National Health Accounts for 2017-18.^8^

#### Additional private household health expenditures

These include additional private expenditures for the national digital health mission (assumed to be 1% of total health expenditure in the state), IPHS/accreditation and quality of care expenditure (assumed to be 2% of inpatient cost), UHC fund for additional care and comprehensive benefits package (assumed to be 1% of total government health insurance expenditure), emergency and pandemic care fund (assumed to be 2% of total health expenditure in the state).

### Role of the funding source

The Christian Medical College Vellore supported the second author (Sudheer Kumar Shukla) through a grant from the 10.13039/501100007296Infosys Foundation. Neither of these two entities had any role in the study design, data collection, data analysis, interpretation, writing of the manuscript, or the decision to submit it for publication.

## Results

### The outside-in approach

**Fig. 1:**
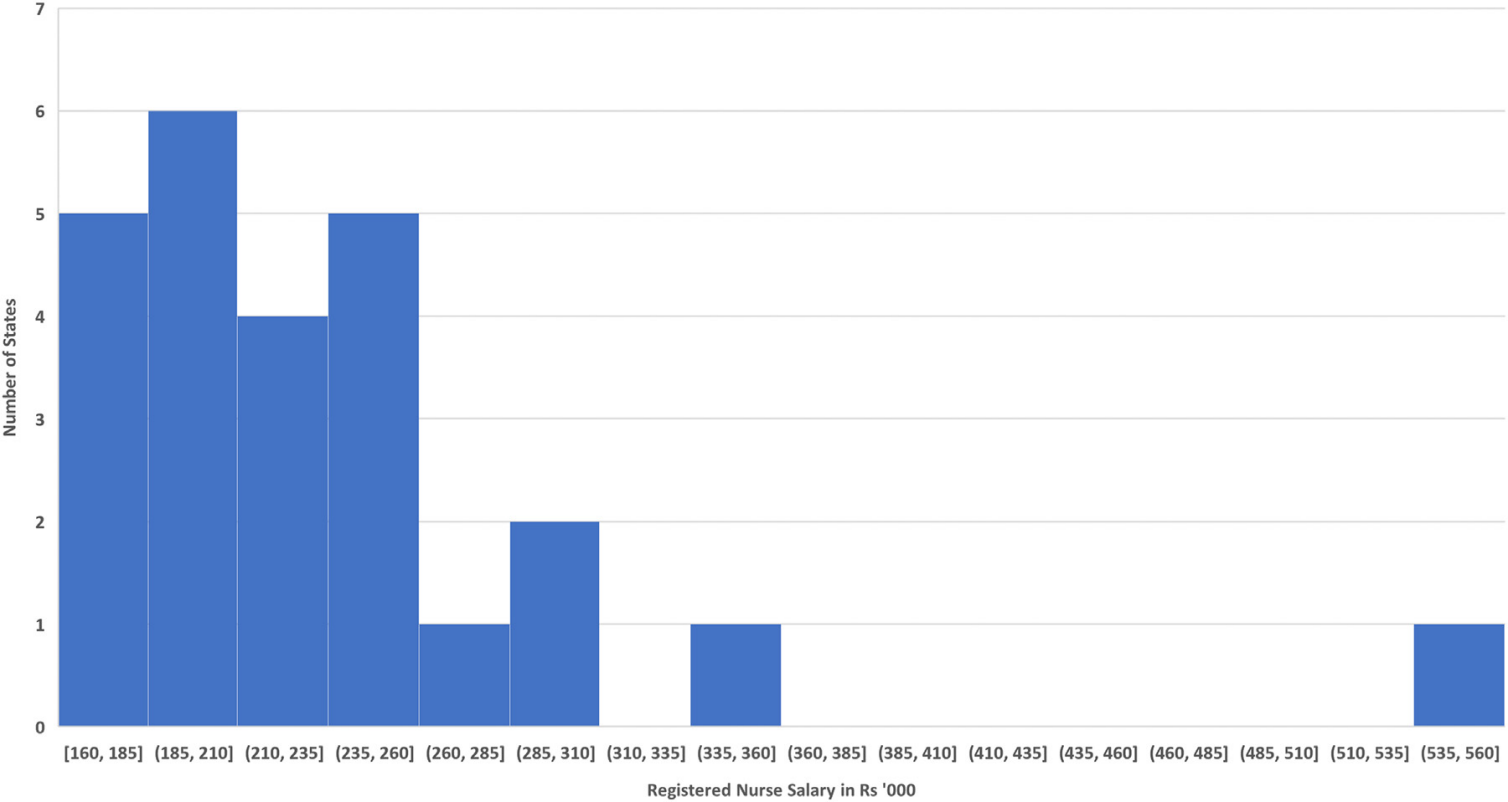
Distribution of registered nurse salaries.

As mentioned above, there are large differences between the per capita health expenditures of state governments, with none of the states offering UHC to its residents. The idea that cost differences between states could drive these is difficult to reconcile with the notion that India is a single common market for healthcare personnel and services. Nursing salaries in India for each state are given in Fig. 1 (see underlying data on nursing salaries in the UHC Costing Data Supplement Outside In Approach). After excluding outliers from the union territory of Puducherry and Sikkim (India's least populous state), it can be seen that there is very little variation in these salaries between states. One implication of this is that the government health expenditures of each state should not be compared with the per capita income of each state but instead with the per capita GDP of the entire country. This represents an important shift in the understanding of relative proportions allocated to health between states, but it still does not tell us if any of these amounts are adequate for the provision of UHC.

To arrive at an understanding of the sufficiency of these expenditures using an *outside-in* approach, it becomes important to arrive at an estimate of the equivalent US$ value of the amounts spent by each state using an appropriate exchange rate, to allow its proper comparison with the expenditures incurred by other countries. As discussed earlier, these exchange rates are referred to as *Purchasing Power Parity* (PPP) exchange rates.^20^ The International Monetary Fund publishes an overall PPP$ for each country based on a comparison of the local and US$ prices of a predetermined basket of goods and services.^20^ For India, this exchange rate is 22.59 per US$^20^ against the prevailing market exchange rate of close to 79.91 per US$ (rates prevailing at the close of business on 22 July 2022,^29^). However, given the high labour intensity in healthcare, perhaps a more appropriate exchange rate to use would be one that is based on the relative salaries of healthcare personnel in the US and in India:

Equation (1) $${PPP\$}_{Health}=\frac{Indian\;health\;worker\;salary}{US\;health\;worker\;salary}$$

India, due to its strong capacity in graduate education and its large workforce, unlike many other developing countries, has an unusually favourable cost/talent ratio. For example, nursing salaries in India are close to 200,000 per year (see underlying data on nursing salaries in the UHC Costing Data Supplement Outside In Approach) while similarly-trained nurses in the United States are paid an average of US$ 77,460.^30^ If these salaries of nurses are used to estimate it, Equation (1) becomes as follows.

Equation (2) $${PPP\$}_{Health}=\frac{Rs.\;200,000}{\$77.460}=Rs.2.58/US\$$$

In Table 1, an implied PPP$ exchange rate associated with the total costs of different procedures in the US and India is arrived at (for detailed computations, see UHC Costing Data Supplement Outside In Approach). It is interesting to note from Table 1 that even in the case of advanced tertiary care operations that are likely to be significantly more capital intensive than primary care, the implied exchange rate ranges from 4.49/$ to 16.98/$ and in all cases is below the IMF's PPP$ exchange rate of 22.59 per US$.^20^ These numbers are also consistent with other analyses that arrive at implied exchange rates of 2.59/$ for heart valve replacement, 2.89/$ for heart bypass, 11.19/$ for hip replacement,^31^ 10.53/$ for cancer, 14.37/$ for kidney dialysis,^32^ and between 4.51/$ to 7.67/$ for coronary artery bypass graft surgery (CABG).^33^

The value of 2.58/$ arrived at in Equation (2) and the range of numbers in Table 1 suggest that an exchange rate of 5/$ (author estimate) could be a more appropriate one to use when converting Indian healthcare costs to US$ equivalents, than the PPP$ rate of 22.59/$ provided by the IMF,^20^ to facilitate global comparisons with other countries.

Table 2 (for detailed computations, see UHC Costing Data Supplement Outside In Approach) first compares the GHE of each state with its GSDP as well as with the national GDP. It finds that while a state like Bihar may be spending 1.4% relative to its own GSDP as a proportion of national GDP, its expenditure is a mere 0.4%. And for a state like Kerala, while its GHE is only 1.1% of its own GSDP, it amounts to a much larger 1.7% of the national GDP. This suggests that if indeed the required government expenditure for UHC in each state is similar on a per-capita basis across states, a state like Bihar may be much further away from it than would appear to be the case if one merely compared ratios of these expenditures to GSDPs for different states.

In Table 2, additionally, government health expenditure in each state is converted to PPP$ using exchange rates ranging from 2 to 80 (for detailed computations, see UHC Costing Data Supplement Outside In Approach). This analysis suggests that with exchange rates between 2 and 5, given the substantial Indian cost advantages, almost all Indian states spend well above PPP$200 per capita. This, of course, raises the question of why, despite this, India and especially states such as Kerala and Himachal Pradesh and many states in the north-eastern region, whose government health expenditures are above PPP$400 per capita, have not moved closer to UHC already, when Thailand was able to do so in 2004 with a per capita government health expenditure at that time of only PPP$193.64.^34^

A cross-country study found that while it is possible to provide better healthcare services at all levels of healthcare expenditure, “in terms of financial protection, significant improvement is observed across our sample of countries only once public spending is greater than PPP$200 per capita”.^35^ Developing countries, such as Colombia, Jordan, Nicaragua, Peru, and Thailand, have built high-performing health systems with government health expenditures in the neighbourhood of PPP$400 per capita per year (see Tables 4 and 5 in Mor^10^). With these global benchmarks in mind, using a PPP$ exchange rate of 5/$, it can be argued that:

(Equation 3) $$PPP\$100<\;GHE\;<\;PPP\$275\equiv 0.25\%<\frac{GHE}{National\;GDP}\;<1.00\%\equiv Basic\;Healthcare$$

(Equation 4) $$PPP\$275<\;GHE\;<\;PPP\$400\equiv 1.00\%<\frac{GHE}{National\;GDP}\;<1.50\%\equiv ModerateUHC$$

(Equation 5) $$PP\$400<\;GHE\equiv 1.50\%\;of\;GDP<\frac{GHE}{National\;GDP}\equiv Full\;UHC$$

where,

(Equation 6) BasicHealthcare≡EPHFProvision+SDOHPolicy+SecondaryProvision

(Equation 7) ModerateUHC≡BasicHealthcare+TertiaryInsurance

(Equation 8) FullUHC≡ModerateUHC+PrimaryCareFinancing

The classification of healthcare services into basic healthcare, moderate UHC, and full UHC used here takes the view that the first priority for any government (basic healthcare) is close attention to policies that impact the social determinants of health (SDOH) and the full performance of all the essential public health functions and services (EPFH/S). Within the ambit of basic healthcare, these need to be complemented with universal access to high-quality free secondary care, strong governance of primary care that multiple providers are already offering, and the establishment of a strong digital backbone to interconnect all of these multiple providers, including components such as electronic health records, computerised decision support, tele-ICU services, and automated, low-cost payment systems. The next step (moderate UHC) would be to offer universal financial protection through insurance tools against very high-cost and rare conditions, such as cancer and advanced cardiac surgeries. Financing primary care through direct free provision or payment to private providers would be the final step in this journey towards full UHC. Also, see Mor^36^ for a more detailed discussion on this.

Using this taxonomy, it can be seen from Table 2, Table 3 that there are several states that have the potential to offer *Full UHC* and *Moderate UHC* to their residents. This analysis suggests that at 2000 per capita, states should be able to offer full UHC to all their residents.

### The actuarial approach

Assuming that the state-level differentials are negligible, another potential way to estimate the UHC costs is to use actuarial methods with simulated data shown in Table 4 (for detailed calculations, see UHC Costing Data Supplement Actuarial Approach). The simulations suggest that even at the high end, UHC costs are expected not to exceed 1600 per capita, a number even lower than the 2000 estimated above.

As mentioned earlier, a significant limitation of this approach is that there are no formal sources for the data used in Table 4. Some confidence in the estimate can, however, be garnered from the fact that the Pradhan Mantri Jan Arogya Yojana (PMJAY, the flagship tax-funded hospital insurance scheme of the government of India,^37^) has set an upper limit of 1500 per family (of five) per year,^38^ implying a per-capita hospitalisation insurance premium of 300 per year. If this number is doubled to account for the coverage of primary care expenditures (the UK National Health Service, which runs a mature, primary care-focused health system, spends 51% of its total annual health expenditure on non-hospital-based services,^39^) the resultant 600 per year is close to the 705 estimated in the mid scenario in Table 4. It is also noteworthy that in all three scenarios in Table 4, primary care costs account for about half the total UHC costs (ranging from a high of 50% to a low of 40%). Table 5, which provides an analysis of 2 million PMJAY claims that were filed from the initiation of the scheme until May 15, 2019,^12^ also provides indirect support for the revenue and incidence rate assumptions used in Table 4.

Commercial insurance prices also offer interesting benchmarks. Prices for the highest-rated comprehensive health insurance plans (for a 5 lac cover) range from 15,808 to 19,374 for a family of four,^23^ i.e., 3952 to 4844 per capita. However, on account of the high distribution and administrative costs, the “incurred claim ratio” for commercial insurers is as low as 60% even for a large insurer like SBI General Insurance,^40^ indicating that only about 60% of the insurance premium, i.e., 2371 to 2906, can be used as a benchmark for computing UHC costs. While these are higher than the estimates arrived at in Table 4, they are of a similar order of magnitude, and a significant portion of the difference could be accounted for by the higher rates charged by private healthcare providers and the distortions in the utilisation patterns embedded in commercial insurance contracts.

### The normative approach

Starting with facility-level data, the human resource, consumables, and infrastructure expenditures associated with the IPHS 2022 norms have been computed. The total costs of UHC with the normative approach, using the IPHS 2022 norms and the 2022 costs, are reported in Table 6. It can be seen from Table 6 that the per capita cost estimate on a nationwide basis comes out to be 3286 (additional details on the data and the computations can be found in the UHC Costing Data Supplement Normative Approach).

### The inside-out approach

Based on the data and the methods discussed earlier, the results shown in Table 7 have been obtained. The estimated total required using this approach is 5552 per capita (additional details on the data and the computation methods may be obtained from UHC Costing Data Supplement Inside Out Approach). The amount of 5552 per capita is significantly higher than any of the other estimates obtained through the other methods, suggesting that it may well be indicative of the extent of inefficiency embedded in the current health systems design and expenditure patterns rather than a reasonable estimate of the cost of providing UHC in India.

In a state like Kerala, for example, where GHE at 2272 per capita per annum is more than 2000, some aspects are strongly indicative of inefficiency in resource use. These include the continued over-use of the C-Section as a procedure by the public sector at a growing rate, from a high of 31.4% in 2015–16 to 37.2% in 2019–20, with, in some districts, such as Ernakulam, Palakkad, and Thrissur, the public sector rates exceeding the already very high rates in the private sector,^41^ even after a 2014 review of maternal deaths in Kerala pointed out that there were a “number of deaths due to postpartum sepsis, especially following caesarean section”.^42^ Additionally, even though it has one of the highest NCD burdens in the country (see page e1343 of Prabhakaran and colleagues^43^), it has low utilisation of public sector primary care facilities which, in Kerala, are well equipped and on which high levels of expenditure are being incurred.^44^

## Discussion

Table 8 gives the UHC estimates arrived at using the four different approaches examined here and in other similar attempts. The average Indian inflation rate for the period 2012–2022 is 5.25%, ranging from 3.33% in 2017 to 10.02% in 2013.^45^ After adjusting all the estimates at a uniform inflation rate of 5% with 2018 as the reference year, all approaches give an estimate of between 1302 and 2703 per capita for UHC, with the exception of the *Inside-out* approach. This suggests that several Indian states may have an inherent ability to offer UHC with government financing alone and that a high degree of waste and inefficiency in the manner in which government funds are being deployed may be behind their apparent inability to do so already. The very high C-section rates provide some indications of this within the public sector in the southern states^46^ and the very low utilisation of government-provided primary care in multiple states.^44^^,^^47^, ^48^, ^49^

Another implication of these results is that several states may be further away from the goal of offering UHC than an initial analysis of their GHE/GSDP ratios may suggest. Of particular concern are the states of Bihar, Jharkhand, Madhya Pradesh, and Uttar Pradesh, all of which have GHE/GSDP >1%, but because their absolute levels of GHE are well below 2000, in order to reach UHC, they may need to triple or, in the case of Bihar, even quadruple their annual health budgets (Table 9). Across the country, using the GHE per capita target of 2000, there is a total shortfall of 103,708 in aggregate GHE to get to UHC (Table 9; for detailed computations, see UHC Costing Data Supplement Outside In Approach). This shortfall ranges from 44,023 to 230,000 as the per capita GHE target is varied from 1500 to 3000 (Table 10; for detailed computations, see UHC Costing Data Supplement Outside In Approach).

### Conclusions

There is a wide variation in the amounts spent per capita by different state governments on healthcare, ranging from 556 for Bihar to 9450 for Arunachal Pradesh (see Table A7 in National Health Accounts for 2017–18^8^) and yet none of the states offer UHC to their residents. The analysis in this paper suggests that given the substantive cost advantages that India enjoys in human resources and on multiple other fronts, 2000 per capita (at 2018 prices) may be a reasonable estimate of the amount needed to offer UHC (see Table 8). Using this as the target for per capita government expenditures on health, it is clear that there are several states that spend far more than would be needed to offer UHC, and their inability to offer it may be related more to the inefficiency and ineffectiveness with which they allocate these resources than to the paucity of funds (Table 9). However, there are also several other states that need, potentially with significant additional support from the central government, to triple or even quadruple their health expenditures to have sufficient funds to offer UHC. Meanwhile, they need to focus their attention urgently on the prioritisation of their current expenditures to ensure that even the limited funds that they currently allocate to health have the maximum possible impact on the welfare of their residents.

### Limitations

There are several limitations associated with the analysis presented in this paper. Perhaps the most important one is that given the current very different epidemiological and demographic profile for each state,^50^ it is entirely possible that even if the base costs are identical, the manner in which the resources need to be deployed could differ quite a bit and the single estimate of per capita GHE of 2000 may not be accurate. However, all Indian states are experiencing a rapid epidemiological transition and appear to be gradually converging, albeit at different rates, to a situation where the burden of non-communicable diseases and injuries (such as self-harm) is likely to become the dominant contributors to the state's burden of disease.

The other limitation is that in the Normative approach point estimates of costs associated with each input have been used instead of the range that would be visible in the market if one were to actually go about implementing such an approach. This limitation is partially addressed through the use of other three approaches each of which provides different estimate with one, the Actuarial approach, also providing a range. These estimates along with the two older ones discussed above, provide a range of values within which the *true* cost associated with UHC is expected to lie.

## Contributors

The first author (Nachiket Mor) contributed to conceptualization, data curation, formal analysis, funding acquisition, investigation, methodology, project administration, resources, software, supervision, validation, visualization, writing – original draft, and Writing – review & editing. The second author (Sudheer Kumar Shukla) contributed to data curation, formal analysis, investigation, methodology, software, validation, visualization, writing – original draft, and writing – review & editing.

## Data sharing statement

All data used in this analysis can be freely accessed both from the paper and the four supplements listed below:

1. UHC Costing Data Supplement Outside In Approach
2. UHC Costing Data Supplement Actuarial Approach
3. UHC Costing Data Supplement Normative Approach
4. UHC Costing Data Supplement Inside Out Approach

## Declaration of interests

Both authors have no conflicts of interest to declare.

## Appendix A Supplementary data

UHC Costing Data Supplement Outside In Approach

UHC Costing Data Supplement Normative Approach

UHC Costing Data Supplement Inside Out Approach

UHC Costing Data Supplement Actuarial Approach
